# Hedgehog/Gli promotes epithelial-mesenchymal transition in lung squamous cell carcinomas

**DOI:** 10.1186/1756-9966-33-34

**Published:** 2014-04-24

**Authors:** Dongsheng Yue, Hui Li, Juanjuan Che, Yi Zhang, Hsin-Hui K Tseng, Joy Q Jin, Thomas M Luh, Etienne Giroux-Leprieur, Minli Mo, Qingfeng Zheng, Huaiyin Shi, Hua Zhang, Xishan Hao, Changli Wang, David M Jablons, Biao He

**Affiliations:** 1 Department of Lung Cancer, Lung Cancer Center, Tianjin Medical University Cancer Institute and Hospital, Tianjin 300060, China; 2 Key Laboratory of Cancer Prevention and Therapy, National Clinical Research Center of Cancer, Tianjin 300060, China; 3 Department of Surgery, Thoracic Oncology Program, Helen Diller Family Comprehensive Cancer Center, University of California, San Francisco, CA 94115, USA; 4 Department of Oncology, Beijing Friendship Hospital of Capital Medical University, Beijing 100050, China; 5 Department of Thoracic Surgery, Xuanwu Hospital, Capital Medical University, Beijing 100053, China; 6 School of Life Sciences, Tsinghua University, Beijing 10084, China; 7 Key Laboratory of Carcinogenesis and Translational Research (Ministry of Education), Thoracic Surgery II, Peking University Cancer Hospital & Institute, Beijing 100142, China; 8 Department of pathology, Chinese PLA General Hospital, Fu-xing Road #28, Beijing 100853, China

**Keywords:** Sonic Hedgehog, Gli, EMT, Lung squamous cell carcinoma

## Abstract

**Background:**

Squamous cell carcinomas (SCC) account for approximately 30% of non-small cell lung cancer. Investigation of the mechanism of invasion and metastasis of lung SCC will be of great help for the development of meaningful targeted therapeutics. This study is intended to understand whether the activation of Hedgehog (Hh) pathway is involved in lung SCC, and whether activated Hh signaling regulates metastasis through epithelial-mesenchymal transition (EMT) in lung SCC.

**Methods:**

Two cohorts of patients with lung SCC were studied. Protein expression was examined by immunohistochemistry, Western blot, or immunofluorescence. Protein expression levels in tissue specimens were scored and correlations were analyzed. Vismodegib and a Gli inhibitor were used to inhibit Shh/Gli activity, and recombinant Shh proteins were used to stimulate the Hh pathway in lung SCC cell lines. Cell migration assay was performed *in vitro*.

**Results:**

Shh/Gli pathway components were aberrantly expressed in lung SCC tissue samples. Gli1 expression was reversely associated with the expression of EMT markers E-Cadherin and β-Catenin in lung SCC specimens. Inhibition of the Shh/Gli pathway suppressed migration and up-regulated E-Cadherin expression in lung SCC cells. Stimulation of the pathway increased migration and down-regulated E-Cadherin expression in lung SCC cells.

**Conclusions:**

Our results suggested that the Shh/Gli pathway may be critical for lung SCC recurrence, metastasis and resistance to chemotherapy. Inhibition of the Shh/Gli pathway activity/function is a potential therapeutic strategy for the treatment of lung SCC patients.

## Background

Lung cancer is the leading cause of cancer-related deaths worldwide. 85% of lung cancer is non-small cell lung cancer (NSCLC), with adenocarcinoma (AD) and squamous cell carcinoma (SCC) as the two major pathological subtypes [1]. Lung SCC is closely associated with tobacco smoking, and it accounts 35% of NSCLC, causing an estimated 400,000 deaths per year worldwide [2]. While recent improvements in targeted therapies such as the EGFR tyrosine kinase inhibitors (TKI), bevacizumab and ALK inhibitors have significantly benefited patients with AD, the effectiveness of these treatments are unfortunately disappointing for lung SCC [3]. Lung SCC patients suffer from poor prognosis with significant rates of reoccurrence and metastasis, largely due to the differences in genetic profiles [4]. Recent studies identified potentially actionable genetic abnormalities in lung SCC, such as phosphoinositide 3-kinase (PIK3CA) amplification, fibroblast growth factor receptor 1 (FGFR1) amplification, and discoidin domain receptor 2 (DDR2) mutation. However, significant efforts are still needed to help in the investigation of the biological characteristics of lung SCC in order to decipher and the mechanism underlying the invasion and metastasis of lung SCC.

Epithelial–mesenchymal transition (EMT) was originally characterized during embryonic development. The concept that EMT being a critical event in the invasion, progression and metastasis of epithelial cancers is well established [5,6]. The molecular basis of EMT involves multiple changes in expression, distribution, and/or function of proteins, i.e. E-cadherin, and the process of EMT is regulated by many molecular events including multiple signaling pathways in various cancers [5]. Furthermore, acquisition of the features of the EMT has been associated with poor prognosis and chemo-resistance, which may allow for recurrence and metastasis to occur after treatment with a standard chemotherapeutic treatment [7-10]. The mechanistic study of EMT regulation could contribute to our understanding of recurrence and metastasis in cancer.

Activation of Hedgehog (Hh) signaling has been implicated in tumorigenesis and metastasis in various cancer types [11-23]. Hh signaling is orchestrated by two trans-membrane receptors, Patched (Ptch) and Smoothened (Smo). In the canonical Hh pathway, in the absence of the Hh ligand, Ptch inhibits Smo, causing cleavage of Gli to the N-terminal repressor form. Once Hh binds to Ptch, the inhibitory effect on Smo is released, causing active full-length Gli to transport into the nucleus and activate transcription of Hh target genes in a context- and cell-type specific manner. Moreover, several studies have revealed "non-canonical Gli activation" in many cancer cell types by which Gli is activated independent of Hh/Smo regulation [12,14]. It needs to be elucidated if the canonical Hh pathway or the non-canonical Gli activation is involved in lung SCC, and if Gli activation contributes to the regulation of metastasis.

Studies of EMT regulation by Hh pathway have recently emerged in literature; data, however, is rare and controversial. While Alexaki et al. [24] and Inaguma et al. [25] suggested that Gli-factor facilitates cancer cell migration and invasion through E-Cadherin in melanoma and pancreatic cancers, Joost et al. [26] proposed that inhibition of Gli promoted EMT in pancreatic cancers. Our study intends to extend the research to lung SCC to help us better understand the regulation of EMT by Hh signaling.

We reported the activation of Hh signaling in two cohorts of patient samples, and revealed the reverse association between Gli1 expression and the expression of EMT markers. Inhibition of the Shh/Gli pathway suppressed migration and up-regulated E-Cadherin expression in lung SCC cells. Stimulation of the pathway increased migration and down-regulated E-Cadherin expression in lung SCC cells.

## Materials and methods

### Tissue specimens

Tissue specimens of the UCSF cohort were collected from 14 patients who underwent surgical resection for lung SCC at the Thoracic Oncology Program at UCSF. Tissue specimens of the Tianjin cohort were collected from 177 patients who underwent surgical resection for lung SCC at the Tianjin Medical University Cancer Institute and Hospital. Samples were fixed in formalin and embedded in paraffin to make tissue slides. The study with UCSF patient tissues was approved by the Committee on Human Research (CHR approval number: H8714-11647-10) at the University of California, San Francisco (UCSF), and that with Tianjin cohort was approved by the Tianjin Medical University Cancer Institute and Hospital. Written, informed consent was obtained from each patient before specimen collection.

### Immunohistochemistry (IHC), immunofluorescence (IF) and Western blot

Immunohistochemistry, immunofluorescence and western blot were performed following standard procedures. Antibodies applied to detect protein expressions in IHC and IF were Gli1 (sc-20687 Santa Cruz Biotechnology, Santa Cruz, CA) at 1:100, Shh (ab 50515 Abcam, Cambridge, MA) at 1:100, Smo (ab 72130 Abcam) at 1:200, Ptch1 (Santa Cruz that Biotechnology,) and E-Cadherin (EMD Millipore) Smo (Sigma, St. Louis, MO) at 1:100, E-cadherin (sc-7870, Santa Cruz Biotechnology) at 1:100, and β-catenin (BD Biosciences, San Jose, California) at 1:400. Antibodies used in Western blot were Gli (C68H3, Abcam) at 1:1000, E-Cad (HECD-1 MED Milliopore, Darmstadt, Germany) at 1:1000 and Actin (A5441, Sigma) at 1:5000. Total protein extraction was performed with M-PER Mammalian Protein Extraction Solution (Thermo Scientific, Waltham, MA), and 40ug of proteins were analyzed in Western blot.

### Cell culture, drug treatment and migration assay

Human lung SCC cell lines H2170, H1703, H1869 and SK-MES-1 were purchased from the Cell Culture Core Facility at Harvard University (Boston, MA, USA). The cell lines were cultured in RPMI 1640 (Life Technologies, Carlsbad, CA) supplemented with 10% fetal bovine serum (FBS) and antibiotics. Cells were seeded one day before treatment with Gli-I and vismodegib (Selleck) at different concentrations and Shh recombinant proteins (eBioscience) for 30 hours, with vehicle (DMSO) as controls. Cells were subjected to the following analyses of immunofluorescence and migration assay. In migration assays, four wounds were made in each condition, and cell migration was presented by the average of distance differences between 30 hr and 0 hr. All experiments have been conducted for more than three times, and representative results were included in the text.

### Statistical analysis

Kappa test was used to evaluate the association between the expressions of Hh pathway components and EMT markers, and between Gli1 and recurrence/metastasis. IHC scores of 1–3 were grouped as positive “+” , and 0 was grouped as negative “-” for dichotomized analysis. Non-parametric Kendall’s tau-b statistics was used to determine the correlation between IHC staining of Hh components. Two-sided student’s t-test was performed for migration assays. A p value <0.05 was indicated as *, 0.01 as **, and 0.001 as *** in corresponding figures. Data analysis was performed using SPSS 17.0 software.

## Results and discussion

### Aberrant activation of the Shh pathway in lung SCC

**Figure 1:**
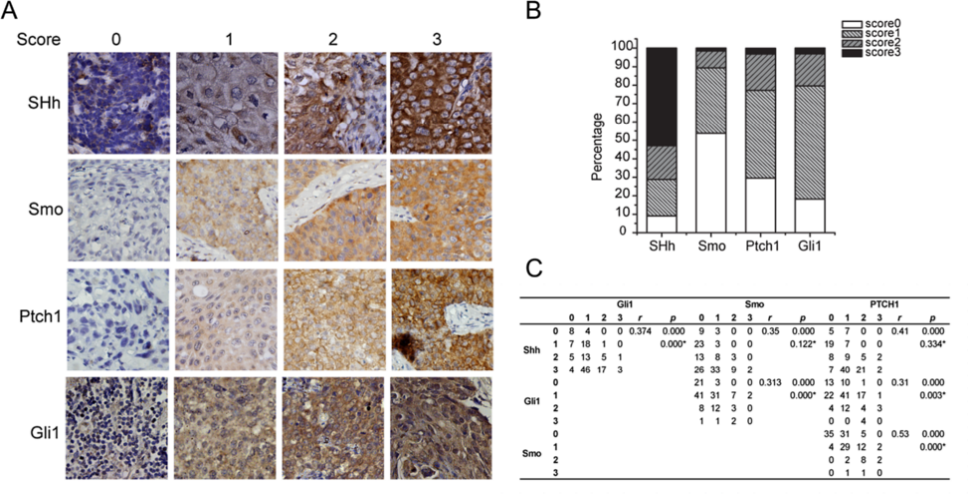
**Aberrant activation of the Shh pathway in lung SCC. (A)** Representative protein expression of Shh, Smo, Ptch1 and Gli1 by IHC staining, scored as 0 (negative), 1 (mild positive), 2 (positive), and 3 (strong positive). **(B)** Expression distributions of Shh, Smo, Ptch1 and Gli1 in 177 patient tissue specimens in the Tianjin cohort. **(C)** Association between the expressions of Hh pathway components. Kendall’s tau-b statsitcs was used to determine the correlation between proteins. The correlation coefficient *r* and *p* values were presented in **(C)**. Kappa test was also performed with IHC scores of 1–3 grouped as “+”, 0 as “-”. Kappa test’s *p* values were labeled with*.

We first investigated the protein expression of key Shh pathway components in lung SCC tissue samples. Formalin-fixed, paraffin-embedded (FFPE) tissue specimens were collected from 177 lung SCC patients who underwent surgical resection at the Lung Cancer Center at Tianjin Medical University Cancer Institute and Hospital. The protein expressions of Shh, Smo, Ptch1 and Gli1 were characterized by immunohistochemistry (IHC), and scored on a scale of 0–3 (negative, mild positive, positive, and strong positive). Representative samples in each score category were summarized in Figure 1A. More than 90% of the lung SCC tissue samples examined were positive for the signal molecule Shh, while 53% and 61% were positive for downstream components and transcriptional targets Ptch1 and Gli1 respectively (Figure 1B). Previous studies have demonstrated limited expressions of Shh components in normal lung tissues at the mRNA and protein levels in NSCLC [27,28], therefore the expression of key Shh signaling components indicates the activation of Shh pathway. No significant association was found between expressions of Shh, Smo, Ptch1 and Gli1 and patients’ characteristics (sex, age, tumor size, or degree of tumor differentiation) (Table 1) (*P* > 0.05, data note shown).

**Table 1:** Characteristics of the lung SCC patients (Tianjin cohort)

**Characteristics**	**No**	**Percent**
**Age (Years)**		
<60	71	40.1%
≥60	106	59.9%
**Gender**		
Male	151	85.3%
Female	26	14.7%
**Smoking history**		
Never	29	16.4%
Smoker	148	83.6%
**Surgical Procedure**		
Lobectomy	143	80.8%
Pneumonectomy	30	16.9%
Extend	4	2.3%
**T stage**		
T1	45	25.4%
T2	107	60.5%
T3	25	14.1%
**N stage**		
N0	126	71.2%
N1	16	9.0%
N2	35	19.8%
**TNM Stage**		
I	91	51.4%
II	48	27.1%
IIIA	38	21.5%

Next we analyzed the association between expressions of key components in the Shh pathway. Kendall’s tau-b correlation tests yielded significant correlations between every two factors (*p =* 0.000), while Kappa’s test suggested strong positive association between SHh and Gli1(*p =* 0.000) (Figure 1C), suggesting the canonical Shh pathway is activated in lung SCC. These data are consistent with previous reports that the upstream Shh signaling has correlations with downstream targets in NSCLC [29,30]. Taken together, our results suggest that aberrant activation of the Shh pathway plays an important role in lung SCC.

### Gli expression reversely correlates with EMT markers

**Figure 2:**
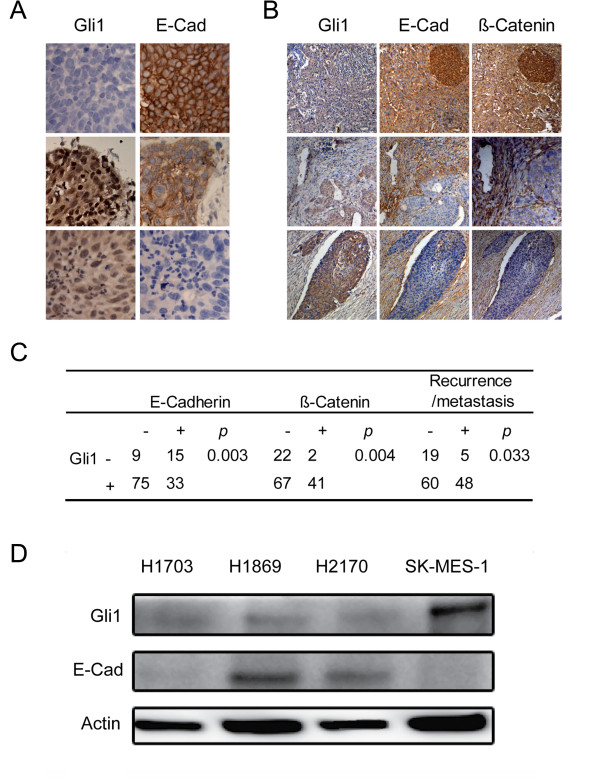
**Gli1 expression reversely correlates with E-Cadherin expression in lung SCC. (A)** Expressions of Gli1 and E-Cadherin (E-Cad) in three representative tissue specimens in the UCSF cohort with Gli1 expression at a low level (upper panels) and high levels (middle and lower panels). **(B)** Expressions of Gli1, E-Cad and β-Catenin (β-Cat) in three representative tissue specimens in the Tianjin cohort with Gli1 expression at a low level (upper panels), a mixed expression pattern (middle panels) and a high level (lower panels). **(C)** Correlations between Gli1, EMT markers, and recurrence/metastasis. Statistical analysis was performed between Gli1 and E-Cad, Gli1 and β-Cat, Gli1 and recurrence/ metastasis. **(D)** Gli1 and E-Cad expression in four lung SCC cell lines by Western blots.

E-Cadherin is a well-established EMT biomarker, and its expression has been suggested to be associated with cancer recurrence and metastasis [5]. The expression of β-Catenin also serves as a biomarker for EMT [31]. To investigate whether the Shh/Gli signaling plays a role in EMT regulation in lung SCC, we first examined 14 lung SCC patients who underwent surgical resection for lung SCC at the Thoracic Oncology Program at UCSF. Eight of fourteen samples showed reverse correlation between E-Cadherin and Gli1 expressions (three representative samples were shown in Figure 2A). To confirm the reverse correlation between EMT markers and Gli1 expressions in lung SCC, we further analyzed E-Cadherin and β-Catenin expressions and correlated with Gli1 expression in the Tianjin cohort. Our results revealed strong reverse correlations between Gli1 and E-Cadherin (*p =* 0.003), as well as Gli1 and β-Catenin (*p =* 0.004) (Figures 2B and C). We also observed reverse correlation between Gli1 and E-Cadherin expression at different areas within one sample in multiple cases due to the heterogeneity of tumor cells (Figure 2), further supporting the reverse correlation between Gli1 and EMT marker expressions. Moreover, our analysis revealed that Gli1 significantly correlated with recurrence and metastasis of lung SCC in the Tianjin cohort (*p =* 0.033; Figure 2C). Consistent with the tissue expression analysis, we observed that Gli1 expression reversely correlated with E-Cadherin expression in four human lung SCC cell lines, H1703, H1869, H2170 and SK-MES-1 (Figure 2D). Taken together, our results indicate the essential role of Gli1, a downstream effector of Shh pathway, in enhancing EMT, which in turn promotes recurrence and metastasis in lung SCC.

### Shh/Gli signaling promotes cell migration by down-regulating E-Cadherin expression

To further understand the role of Shh/Gli in EMT regulation in lung SCC, we manipulated the Shh/Gli signaling pathway in lung SCC cell lines to examine its impact on cell migration and E-Cadherin expression. To inhibit the Shh/Gli activity, we applied two small molecule compounds: Vismodegib and a novel Gli inhibitor. Vismodegib (also known as GDC-0449) is a Smo inhibitor recently approved by the U.S. Food and Drug Administration to treat adult patients with basal cell carcinoma [32-35]. Multiple clinical trials are evaluating the use of vismodegib in other types of cancer, in addition to other candidate drugs that targets Hh signaling [32,36]. The novel Gli inhibitor (Gli-I) developed by our lab specifically inhibits Gli1 and Gli2 transcriptional activity [28]. To stimulate the pathway, we applied recombinant Shh proteins.

**Figure 3:**
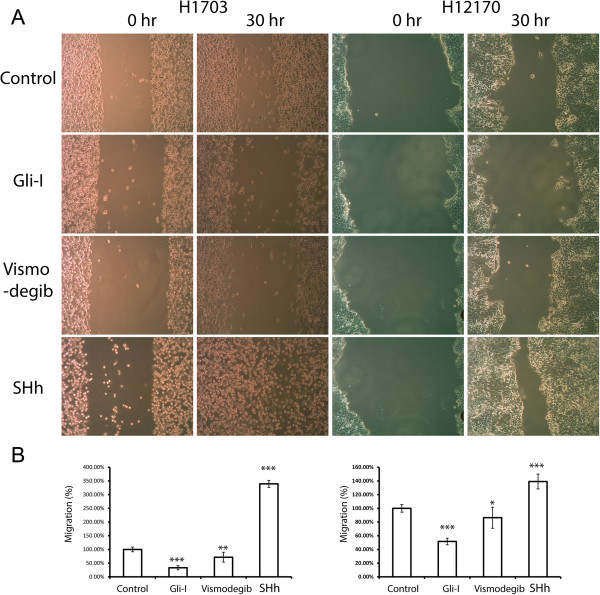
**Shh/Gli signaling promotes cell migration in lung SCC. (A)** Wound healing assays of lung SCC H2170 cells (left) and H1703 (right) treated with Gli-I, vismodegib, and recombinant Shh proteins. Representative pictures shown at 0 hr and 30 hr were taken under a light microscope (×100). **(B)** Quantification of the wound healing assays. The migration distance of cells was set as 100%. A *p* value <0.05 < 0.01 or <0.001 was indicated as *, ** or *** respectively.

**Figure 4:**
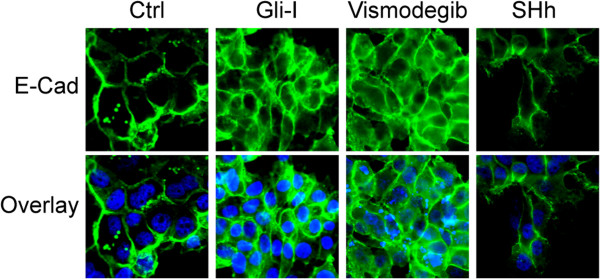
**Shh/Gli signaling down-regulates E-Cadherin expression.** Immunofluorescent staining of E-Cad (green) in lung SCC H2170 cells treated with Gli-I, vismodegib, and recombinant Shh proteins. DAPI (blue) was used to stain nuclei of those cells.

We first performed cell migration assay in lung SCC cell lines H1703 and H2170 after the treatments with either Shh/Gli inhibitors or recombinant Shh proteins. Cells treated with Vismodegib and Gli-I exhibited significantly slower migration in 30 hours; on the other hand, cells stimulated by Shh proteins migrated significantly faster (Figure 3). This data strongly suggests that Shh/Gli signaling plays an essential role in regulating the migration of lung SCC cells. Next we examined E-Cadherin expression in these cells by immunofluorescence staining. We observed that E-Cadherin expression was up-regulated in those lung SCC cells treated with Shh/Gli inhibitors and down-regulated in the cells stimulated by Shh proteins (Figure 4). This is consistent with the mobility of lung SCC cells after the different treatments (Figure 3). Therefore, our results indicate that Shh/Gli signaling may promote cell migration by down-regulating E-Cadherin expression in lung SCC.

## Conclusions

Our study provides evidence for aberrant activation of Shh/Gli pathway and a strong association between expressions of Gli proteins and EMT markers in human lung SCC, as well as the implication of activated Shh/Gli pathway in cell migration and EMT process. Our findings suggest that the Shh/Gli pathway may be a critical component in lung SCC recurrence, metastasis and resistance to chemotherapy. Inhibition of the Shh/Gli pathway activity/function is a potential therapeutic strategy for the treatment of lung SCC patients.

## Competing interests

The authors declare that they have no competing interests.

## Authors’ contributions

DY carried out IHC staining, data analysis, and drafting of the manuscript. HL carried out IF staining, Western blotting, data analysis and drafting of the manuscript. JC, YZ, MM, QZ, and HZ carried out IHC staining and data analysis. HS carried out statistical analysis. HT, JJ, TL, and EG-L carried out the cell cultures and cell-based assays. DMJ participated in the study design and helped to draft the manuscript. CW, XH and BH conceived of the study, and participated in its design and coordination, and helped to draft the manuscript. All authors read and approved the final manuscript.
